# The multiple generator hypothesis of consciousness

**DOI:** 10.1093/nc/niaf035

**Published:** 2025-09-24

**Authors:** Asger Kirkeby-Hinrup, Sascha B Fink, Morten S Overgaard

**Affiliations:** Department of Philosophy, Lund University, Lund, Sweden; Center for Functionally Integrative Neuroscience, Århus University, Århus, Denmark; Centre for Philosophy and AI Research, University of Erlangen-Nürnberg, Erlangen, Germany; Center for Functionally Integrative Neuroscience, Århus University, Århus, Denmark; Department of Neurology, Århus University, Århus, Denmark

**Keywords:** consciousness, neural correlates of consciousness, ncc, theories and models, empirical evidence, methodology

## Abstract

It is well known that in interdisciplinary consciousness studies there are various competing hypotheses about the neural correlate(s) of consciousness (NCCs). Much contemporary work is dedicated to determining which of these hypotheses is right (or the weaker claim is to be preferred). The prevalent working assumption is that one of the competing hypotheses is correct, and the remaining hypotheses misdescribe the phenomenon in some critical manner and their associated purported empirical evidence will eventually be explained away. In contrast to this, we propose that each hypothesis—simultaneously with its competitors—may be right and its associated evidence be genuine evidence of NCCs. To account for this, we develop the multiple generator hypothesis (MGH) based on a distinction between *principles* and *generators*. The former denotes ways consciousness can be brought about and the latter how these are implemented in physical systems. We explicate and delineate the hypothesis and give examples of aspects of consciousness studies where the MGH is applicable and relevant. Finally, to show that it is promising we show the MGH has implications which give rise to novel questions or aspects to consider for the field of consciousness studies.

## Introduction

It is well known that in interdisciplinary consciousness studies there are various competing hypotheses about the neural correlate(s) of consciousness (NCCs). Much contemporary work is dedicated to determining which of these hypotheses is right. The prevalent working assumption is that one of the competing hypotheses is correct, and the remaining hypotheses misdescribe the phenomenon in some critical manner and their associated purported empirical evidence will eventually be explained away. In contrast to this, the multiple generator hypothesis (MGH) proposes that each hypothesis—simultaneously with its competitors—may be right, and its associated evidence be genuine evidence of NCCs. Importantly, the MGH does not claim that ‘every’ available hypothesis about the NCC is correct. The starting point for the MGH is the weaker claim: that that ‘more than one’ hypothesis may be correct. In the context of this analysis, the MGH relates to hypotheses about which principle or mechanism and which neural correlates in the brain that instantiates^1^ this principle or mechanism, and not the metaphysical implications hereof. On this basis, the MGH argues that

1) There may be more than one principle or mechanism, that—independently of each other—‘are sufficient’ for the generation of conscious experience.
2) There may be more than one NCCs, that—independently of each other—are ‘sufficient’ for the generation of conscious experience.

Importantly, as we will show, the MGH does not need any reading of both these claims to be true but needs only (certain interpretations of) either to be true. To lay the foundation for exploring this, in the next section, we will provide a brief introduction to the prevalent contemporary approach in interdisciplinary consciousness studies. This serves as the foundation for introducing two central distinctions to flesh out the MGH in section three. Following this, in section four we show how the MGH is applicable and argue that it is relevant to the field of consciousness studies. Finally, in section five, we show that the MGH is a promising hypothesis by pointing to potential avenues for future research and offering some concluding remarks.

## A pre-theoretical assumption in contemporary consciousness research

Most contemporary theories of consciousness have proposed empirical findings suggesting that the neural correlates of consciousness (NCCs) are found in one or the other brain region. Proponents of the local recurrency theory (LRT) hold that consciousness correlates with recurrent feedback to early sensory regions (Lamme 2004, 2006, 2018). In contrast, proponents of high-order theories (HOT) suggest that the NCCs may be found in the dorsolateral prefrontal cortex, a region supposedly suitable for catering to the special metacognitive representations the theory posits as necessary for consciousness (Lau and Rosenthal 2011; Odegaard et al. 2017; Lau and Brown 2019). Those who advocate the global neuronal workspace theory (GWT) suggest special neurons—primarily in fronto-parietal regions—facilitate the distribution of information they think underpins conscious experience (Baars 2005; Mashour et al. 2020). According to the pre-theoretical assumption, only one of these hypotheses can be right. The same goes for the large number of other theories of consciousness that exist in addition to our examples here.

The extensive contemporary work related to how to assess and compare theories can be seen as a consequence of the pre-theoretical assumption. In other words, because the field is concerned with figuring out who is right, the onus has been on ways to evaluate theories. Examples include recent attention to how we assess and compare theories (Melloni et al. 2021; Melloni et al. 2023; Chis-Ciure et al. 2024; Negro 2024; Kirkeby-Hinrup 2024b), the relation between theory and evidence (Del Pin et al. 2021; Kozuch 2024; Kirkeby-Hinrup 2024), what evidence can and cannot do for us (Fink 2016; Overgaard and Kirkeby-Hinrup 2021), what is required of an explanation (Signorelli et al. 2021; Schurger and Graziano 2022; Kirkeby-Hinrup et al. 2024), and whether some questions are mandatory to answer (Doerig et al. 2020).

Broadly speaking, the general sentiment—given the prevalent view of the mind-brain relationship—is ‘that the way we go about’ figuring out which theory is right, involves empirical sciences. Candidate domains for exactly ‘which’ empirical sciences are relevant include at least neuroscience, cognitive science, biology, physics, psychology, and computer science, and possibly others. The three theories discussed above primarily rely on evidence from the domains of neuroscience, cognitive science and psychology, which are also the predominant empirical domains in the interdisciplinary field.^2^ Interestingly, within these domains, there is some indication that certain theories prefer certain kinds of data or methodology (Yaron et al. 2022).

In the current context, what is interesting are not the details of the empirical support of theories, but rather the fact that they ‘all have’ empirical support. There is something here worth noting: ‘All*’* hypotheses are apparently supported by data. This is not merely a case of theories being underdetermined by the evidence, since theories are supposedly mutually exclusive, and some data speaks for one theory but against another.^3^ If we see evidence for both for some hypothesis ‘and’ its competitors, this suggests an additional challenge: Whatever theory we prefer, it is not sufficient to only give an account of the empirical evidence taken as support for that theory. One also needs to give an account of why there seems to be evidence in favor of its competitors. A common strategy is to argue that the evidence proposed in favor of competing theories is associated with processes that accompany conscious experience (or reports of conscious experience), but which are not true NCCs. They are precursors and/or effects of the true NCC (Tsuchiya et al. 2015). On this view, the neural data only ‘appears’ to be evidence for competing hypotheses but is ‘not actual’ evidence. The expectation is that, eventually, it will turn out that the competing theories misdescribe the phenomenon in some critical manner and their associated purported empirical evidence will be explained away.

As a methodological assumption, the pre-theoretical assumption (i.e. trying to show that one theory is right) is not an ‘a priori’ bad idea. For ease of exposition, let us call this the ‘exclusivity’ approach. Now, given how few approaches there are, and how little we know, the exclusivity approach is justified merely by ‘being a way’ to approach the subject (consciousness). Furthermore, it is possible that the exclusivity approach may yield significant progress in the field. Such success of the exclusivity approach has been seen elsewhere in the history of science.^4^

However, in the case of consciousness studies, if the exclusivity approach is rooted in the pre-theoretical assumption, it is inherently optimistic and uncertain. It is ‘optimistic’ because it expects the world to be in a certain way; specifically a way where only the exclusivity approach is applicable. It is ‘uncertain’ because there are difficulties with determining whether the exclusivity approach itself is applicable and feasible in the first place (c.f. the above). The existence of alternative ways the world might be (e.g. if illusionism, panpsychism, or the MGH is right) where the exclusivity approach is not applicable puts pressure on the exclusivity approach with respect to justifying adhering to the pre-theoretical assumption. To illustrate, if the world is such that the MGH is true, then any conclusion from NCC evidence that one theory is right and others are wrong (following the pre-theoretical assumption) is faulty. Since we do not know how the world is with respect to consciousness, and it is possible that the world might be such that the MGH is true, it becomes difficult to justify only embracing an exclusivity approach rooted in the pre-theoretical assumption. Indeed, if the world is such that the MGH is true, embracing the pre-theoretical assumption would constitute a radical confound in the study of consciousness. Furthermore, as evidenced from the above the exclusivity approach itself faces seemingly systemic issues with how we expect empirical evidence to help determine which theory is right (Cohen and Dennett 2011; Overgaard and Kirkeby-Hinrup 2021; Kirkeby-Hinrup 2024a), which puts further pressure on the exclusivity approach to show that its feasible, let alone that it should is only approach we should pursue.^5^

Our point here is that we should not be blind to alternative approaches just because we have the exclusivity approach. In fact, any alternative approach would be justified by the same reference point as the exclusivity approach, namely the scarcity of approaches. Furthermore, if an alternative approach is incongruent with the exclusivity approach this in fact is a good thing, because together the two approaches cover more possible ways the world could be.^6^

## The multiple generator hypothesis: An alternative approach

The MGH takes its starting point in a rejection of the pre-theoretical assumption and asks: What if more than one hypothesis can be right? The fact that we find evidence for competing theories of consciousness’ respective NCC hypotheses can be taken as initial support for this alternative. According to MGH the fact that we see empirical evidence in favor of more than one of the competing NCC hypotheses is explained by the fact that more than one of these hypotheses is right. This means that on the MGH, there may be multiple locations in the brain each generating consciousness independently. Thus, the competing hypotheses are not mutually exclusive in the sense that one being correct entails that the remaining hypotheses are incorrect (as the pre-theoretical assumption holds).

A hypothesis about the NCC is a proposition about how (some kind of) activity (at some location) in the brain is correlated with conscious experience. Ideally, such a hypothesis should answer ‘what in’ (e.g. neuron populations, brain tissue, electrical signals, activation patterns), ‘where in’ (e.g. brain areas, brain topology, white matter, grey matter), and ‘how’ (some ‘principle’, see below) the brain generates conscious experience. Answering these questions amounts to defining what we will here call a ‘generator’ of consciousness. Given that consciousness exists (and ‘ex hypothesis’ is generated by the brain) it follows that there is—at minimum—one combination of answers to these questions that reflects the way the world is (i.e. how consciousness is instantiated in humans). In other words, there is a set of answers that is correct about at least one existing generator (for an individual to be conscious at all, there has to be at least one generator). The MGH suggests that there may be more than one set of answers.

The work in recent decades (given the pre-theoretical assumption) attempts to deploy the ‘what’ and ‘where’ questions to answer the ‘how’ question, where the answer to the ‘how’ question is assumed to be one of the competing theories. However, it has previously been shown that this strategy is not viable (Overgaard and Kirkeby-Hinrup 2021) because it is not possible to infer from the location of NCC evidence to the principle it instantiates. For this reason, it is necessary to say something more about the relation between ‘generators’ and ‘principles’. When we talk of ‘principles’ we mean it to denote ‘ways conscious states can be generated’.^7^ Consider, as an analogy, the difference between a mechanical clock and a digital one: They are obviously different generators with respect to ‘what’ and ‘where’, and ‘how’, but work along the same principle, namely that a stable progression of something (a stable turn of a gear versus a stable resonance frequency of a crystal) is used to drive the hands on the clockface rhythmically. Consequently, multiple distinct generators using the same principle seems possible. What about cases where there are multiple principles in play? Let us consider the case of heat. Heat is a property associated with the energy of fast moving atoms, but there are many ways of generating this property (principles) such as metabolism, friction, and combustion. This is akin to the MGH idea where a single phenomenon (heat or consciousness) can be generated in multiple ways.

As we saw above, contemporary practices associated the *how* question with specific theories of consciousness and their conceptual frameworks. While the MGH rejects this association, candidates for—and illustrations of—principles can nevertheless be found in extant theories. For instance, the principle invoked in HOT theory is that conscious states (analogous to heat) can come about from a system keeping track of—or representing—its own internal states in a special way.^8^ To give another example, workspace theory holds that consciousness (analogous to heat) derives from the amplification and broadcasting of information across the brain. Each of these two (and similar ones derived from other extant theories) is a proposal of a ‘way consciousness is generated’*.* In the context of MGH, they are proposals of principles. This allows another way to specify the MGH as suggesting that more than one principle exists, i.e. there is more than one way conscious states can be generated. This, in turn, lets us refine the notion of a generator introduced above, because in addition to being defined by what, and where, a generator should instantiate a principle (how).

With the distinction between principles and generators at hand we can map the ways the world could be, as a possibility space consisting of a two-by-two matrix (Table 1), where the first dimension charts whether there is one or multiple principles, *i*.e. ways consciousness can be generated. The second dimension charts whether there is one or multiple generators in the brain, i.e. areas that generate conscious contents independently of each other.

**Table 1:** Legend: Possibility space based on numerosity of generators (rows) and principles (columns)

		Generators	
		Single	Multiple
Principles	Single	A (Single Generator and Single Principle)	B (Multiple Generators and Single Principle)
	Multiple	C (Single Generator and Multiple Principles)	D (Multiple Generators and Multiple Principles)

If one follows the exclusivity approach (given the pre-theoretic assumption that one theory is right) the principle dimension is fixed along the ‘single’ column in this matrix. Consequently, on the exclusivity approach the only live options are A and B. Most theories seem to argue for variants of A, i.e. that there is one principle, and it is instantiated in a single generator (their favored NCC hypothesis). One motivation for this may be a relation of fit between a principle and a given brain area. For instance, so-called cognitivist theories are generally associated with frontal areas because the prefrontal cortex in turn is associated high-level cognition, such as abstract and metacognitive functions (behavior). It is unclear how strong the inference can be made from theory to location, but it is worth noting that (as already mentioned) inferring in the other direction is unviable (Overgaard and Kirkeby-Hinrup 2021) which may give one pause. Be that as it may, there are at least a few theories—such as the local recurrency theory (positing the recurrent processes in early sensory regions underpin conscious experience of a given sensory modality)—that can be seen to argue for B by suggesting there are multiple independent generators (e.g. in the various sensory systems) operating on the same principle (this is also touched on in Overgaard et al. 2024 albeit in the context of sensory interplay). Something similar seems to hold for the REF-framework proposed by Mogensen and Overgaard (2018). Interestingly, the Joint Determination Theory of Biyu He (He 2023) seems to fall somewhere in the middle, as she suggests multiple NCC locations collaborating dynamically based on task demands, suggesting multiple (context dependent) answers to what and where, but without committing to an answer for how. In one sense this is a continuation of the exclusivity approach, where determining what and where is taken as a first step to determine how. A similar idea is found in the construct first approach suggested by Peter Fazekas and colleagues (Fazekas et al. 2024), which suggests to start by solving the what (and partly how) in a theory-neutral way (via the concept of ‘constructs’) to avoid problems they see with the exclusivity (theory-centric) approach evident in contemporary practices.

Returning to the matrix, and our posit in this paper. Clearly, if there is more than one way to generate consciousness, this rules out the possibility of A, in which there is only one principle and one generator. Consequently, in the context of this matrix, there are two different ways the MGH might obtain, corresponding to the multiplicity options along each dimension, giving us either the sets of [B;D] or [C;D]. Let us investigate these in turn. Starting with the possibility that there are multiple generators (giving us the possibilities of B and D), more than one theory can be right, because their answers to where (localization of the NCC) may simultaneously be right. Alternatively, if we fix the multiplicity along the principles dimension (yielding the possibilities of C and D), more than one theory can be right because there is more than one way to generate consciousness (corresponding to hypotheses about how that can be derived from them).

There are many more details to flesh out with respect to the MGH, and each of these may carry interesting upshots worthy of further exploration. We will touch on this again in the concluding remarks below. However, the simple matrix conception of the MGH offered here is sufficient to have interesting consequences and offer novel ways to view, address, or explain some open issues in consciousness studies. We will consider a selection of these in the next section.

## The MGH: Applicability and relevance

In the rest of this paper, we will argue that the MGH is an applicable, relevant, and promising hypothesis. To show that it is applicable involves showing that it can be applied to the subject (consciousness) or field (consciousness studies). If it is not applicable, the point is moot. To show that the MGH is relevant consists in showing that the MGH impacts scientific practices in the field. Relevance matters because, clearly, if the MGH has no scientific consequences, there is no incentive to pursue it. Finally, showing that the MGH is promising involves pointing to potential avenues of future research to demonstrate that it may be worthwhile to put more effort into exploring it. We will postpone discussion of whether the MGH is promising to the next section, and here focus only on whether the MGH is applicable and relevant.

In the previous section we offered a preliminary delineation of the possibility space of MGH according to the numerosity of principles and generators. The purpose of this merely was to establish that there is a hypothesis associated with MGH. With respect to our objective in this section, because the MGH takes its starting point in the empirical evidence proposed in the context of the NCC debates, and offers a novel approach to understanding this evidence, it is clearly applicable to the field of consciousness studies.

To show that the MGH is relevant, we here offer three very different contexts in consciousness studies where the MGH offers a novel perspective: phenomenal variability, non-human consciousness, and scientific methodology. Each of these three contexts treat the phenomenon at very different levels of description and abstraction. These contexts we picked intentionally to illustrate the variety of areas impacted by the MGH. Importantly, the MGHs role in each context deserves lengthy separate treatment. In other words, we appreciate that there are details to be worked out in each context.

Starting with phenomenal variability, one issue in the field consciousness studies appears to be differing intuitions about consciousness between researchers in the field. One possibility is that these intuitions may in fact be grounded in what it is like for the individual researcher to be conscious (how consciousness appears subjectively to the individual), and this seems to vary in substantial and radical ways between individuals (e.g. suppose between eliminativists and phenomenalists). In one paper, Sascha Fink (2018) suggests that perhaps such phenomenal variability may be a consequence of neuro variability. i.e. the fact that no brain is exactly alike could be a reason to think there is variability in the experiences they generate. But it may also be due to variability in what is generating the experiences. This is the case with the MGH where individuals contain a set of generators (c.f. the matrix above, multiple independent implementations of one [B] or more [D] principle). Qua ‘outputting’ experiences, the generators contribute to what it is like to be the individual at a given time. Consequently, phenomenal variability can be explained by appeal to differences in generators.

Similarly, in the context of non-human (biological) consciousness, the perceived differences between (putative) animal consciousness and human consciousness can be explained by differences in their respective sets of generators. One implication of such a difference would be that when assessing consciousness in non-humans we should not require that they contain structures that are isomorphic to human brain organization and function for the obvious reason that their set of generators may differ from that of humans. Similarly, the difference between primate consciousness and non-primate consciousness may be due to the former either implementing a higher number of generators (a quantitative explanation) or implementing principles that the latter does not (a qualitative explanation). Interestingly, these considerations may be similarly applicable to debates about consciousness in non-biological systems (i.e. contemporary AI debates). To elaborate, according to some theories (e.g. HOT) most non-primate consciousness is ruled out by reference to a supposed lack of metacognitive capacities inferred from their less developed prefrontal areas. Conversely, some theories (e.g. LRT or IIT) are less skeptical about non-primate consciousness given that their sensory systems may cater to specific processes or properties. Given that we have some indication that (some) non-primates may be conscious (behavioral evidence making them appear conscious, or the weaker claim that we are less reluctant to ascribe consciousness to them) their lack of metacognitive capacities need not be deployed to rule out consciousness, but rather as an opportunity to explore specific possible generators in isolation (i.e. without confounds from possible human generators in the PFC, or cognitive confounds, c.f. Block 2020).

This speaks to a generalized point, namely that taking the MGH seriously has radical implications for scientific practices in the field of consciousness studies. The reason for this is that the methodologies deployed to search for the NCC may require revision to account for the possibility of multiple generators. Classical subtractive studies can no longer warrant definitive conclusions with respect to the NCC. This may prompt new methodology to experimentally to test for, or rule out, possible generators, when the presence of other generators are possible.

## Concluding remarks

In the previous sections we first showed that the MGH is applicable because deploying the possibility space allowed us to categorize existing theories and sketch alternatives to the pre-theoretic assumption. Secondly, we demonstrated that the MGH is relevant by considering the MGH in three very different contexts. In this section, we argue that the MGH is promising (i.e. that it offers interesting avenues for future research) and offer some concluding remarks.

To argue that the MGH is promising we briefly highlight three additional implications of the MGH that may facilitate avenues of future research. Preliminarily—of course—we do not purport that the list of possible applications of the MGH offered here is exhaustive. Similarly, each of the contexts and upshots considered in here (and in the previous sections) merits separate further elaboration.^9^

The first implication of the MGH we wish to highlight concerns the distribution of consciousness on earth or in the universe. It may appear that, ‘ceteris paribus’, the possibility of multiple generators or multiple principles makes it more probable that at least one (or more) generator can be implemented in non-humans. Put differently, the MGH (ceteris paribus) makes it more likely that humans are not the only conscious entities in the universe, simply because there are more possible ways physical systems can generate conscious states. Furthermore, it is a live possibility that some principles and/or generators can only be implemented in certain physical substrates. Similarly, it is possible that some principles and/or generators can only be implemented in humans, and some principles and/or generators cannot be implemented in humans.

The second implication concerns neurorehabilitation where the MGH may offer novel theoretical perspectives that may eventually inform clinical practices. To give just one example, questions of interest in this regard could concern the possibility of repairing damaged functions or whether damaged generators can be re-created in new tissue. Interestingly, neurorehabilitation reciprocally offers a promising experimental environment for developing and testing the MGH. This, for instance, with respect to the limits of neuroplasticity, the influence of training (e.g. neurofeedback. See Kvamme et al. 2022a; Kvamme et al. 2022b), and scales for subjective reports (Sandberg and Overgaard 2015; Overgaard and Sandberg 2021).

The third implication concerns the status of existing evidence going forward. In the above, we offered considerations about the MGH and its relation to existing empirical evidence. Clearly the few examples we have provided above do not cover every aspect of the field. We view this as an incentive for further exploration rather than an objection. Put differently, we agree that further investigating empirical evidence in light of the MGH is desirable. This goes not only for novel incoming data, but also for the entire back catalogue of evidence in the field. Importantly, we are not here advocating a radical revisionist movement. The purpose here is not a call to arms, but merely to highlight an unexplored area opened up by the MGH. Targeted approaches, such as scrutinizing the COGITATE data (Melloni et al. 2023; Consortium et al. 2025) in light of the MGH are promising (and less labor-intensive) alternatives to full scale revisionism.

## Footnotes

1 Important to state early on is that the MGH should not be conflated with the classical notions of functional roles and material realizers. While our conception of generators is somewhat similar to the notion of material realizers, the notion of principles on the MGH is a significantly more encompassing than functional roles.

2 As exemplified by *e.g.* the ASSC conference programs. (Observe that while Philosophy also has a prominent place at the ASSC, it is not an empirical science, and therefore not relevant in the context above). Mathematics is also not on the list for the same reasons.

3 We are thankful to an anonymous reviewer for highlighting this.

4 And even in cases where theories seem genuinely incompatible (*e.g.* general relativity and quantum mechanics) often scientists are still trying to reconcile them. Reconciliation (one way or another) is also a possibility with respect to consciousness studies, and as will be clear we are not claiming that the field *must* move toward pluralism but rather that it is possible that consciousness may be special in this way.

5 To be clear, our quarrel here is only with the pre-theoretical assumption (theories are mutually exclusive). We are not advocating against the exclusivity approach since this can co-exist with other approaches. To clarify, the relation between the pre-theoretical assumption and the exclusivity approach is asymmetrical. If one embraces the pre-theoretical assumption, this entails the exclusivity approach (and only that), however embracing just the exclusivity approach does not entail rejecting other approaches.

6 One interesting possibility that we will not cover here, but that nevertheless warrants a short clarification, is the possibility that supposedly competing theories in reality address different explananda, and therefore are not mutually exclusive (Mitchell 2002, 2003; Northoff and Lamme 2020; Singhal and Srinivasan 2024; Storm et al. 2024). On the MGH we define experiences in terms of states that have phenomenal properties. This means that conscious states are states with phenomenal properties, where the latter is defined in the classical Nagelian terms (states that it is like something to be in). On this definition it is hard to see that there can be more than one natural kind. Of course one might say that there are different kinds of phenomenal properties, perhaps visual phenomenality is of a different kind than tactile phenomenality, but they are both phenomenality, so they belong to the same natural kind. Importantly, this answer is neutral on the nature of phenomenality and is not threatened even if phenomenality ends up grounding out in physical states (which we a disposed to think). That being said, we do not think the MGH necessitates the ‘single natural kind’ reply, and exploring the MGH in relation to multiple explananda frameworks constitutes an intriguing avenue of future research. We thank two anonymous reviewers for prompting us to think hard about this.

7 Traditionally, the concept ‘multiple realization’ (MR) denotes the idea that consciousness could be realized in different physical substrates (*e.g.* the brain or a silicon chip). While there are similarities between the ideas, the MGH differs from MR in important respects. Whereas MR is an *a priori* posit about the (metaphysical) nature of consciousness, the MGH is an *a posteriori* claim concerning what is *actually* the case regarding human consciousness in the actual world, and as such entails no stance on CMR. However, this does not mean that there are no connections between the two claims. To some extent, if one endorses the possibility of MGH, this would be an (albeit weak) reason also to endorse CMR, because *ceteris paribus* it seems likely that if there are more ways consciousness can be instantiated in human brains, then it increases the chance that one of these ways of instantiating consciousness may be applicable to non-human-brain substrates as well. Be that as it may, what is important to underscore here is that while MGH shares with MR the idea that consciousness can be instantiated in different ways, they differ because the former is an empirical hypothesis about the human brain and the NCCs, whereas the latter concerns the essential nature of consciousness *per se* and the substrates in which it can be instantiated.

8 Whether one cashes out this principle in terms of higher-order representation or in more general terms like metacognition, is unimportant for our present purposes.

9 Indeed, given that this is the first paper on the MGH, we expect that with more work additional areas where the MGH may be deployed to gain new insights will reveal themselves over time.

## References

[ref1] Baars BJ . Global workspace theory of consciousness: toward a cognitive neuroscience of human experience. Prog Brain Res 2005;150:45–53. 10.1016/S0079-6123(05)50004-9 16186014

[ref2] Block N . Finessing the bored monkey problem. Trends Cogn Sci 2020;24:167–8. 10.1016/j.tics.2019.12.012 31987718

[ref3] Chis-Ciure R, Melloni L, Northoff G. A measure centrality index for systematic empirical comparison of consciousness theories. Neurosci Biobehav Rev 2024;161:105670. 10.1016/j.neubiorev.2024.105670 38615851

[ref4] Cohen MA, Dennett DC. Consciousness cannot be separated from function. Trends Cogn Sci 2011;15:358–64. 10.1016/j.tics.2011.06.008 21807333

[ref5] Consortium C, Ferrante O, Gorska-Klimowska U et al. Adversarial testing of global neuronal workspace and integrated information theories of consciousness. Nature 2025;642:133–42. 10.1038/s41586-025-08888-1 40307561 PMC12137136

[ref6] Del Pin SH, Skóra Z, Sandberg K et al. Comparing theories of consciousness: why it matters and how to do it. Neurosci Conscious 2021;niab019. 10.1093/nc/niab019 PMC8372971 34422317

[ref7] Doerig A, Schurger A, Herzog MH. Hard criteria for empirical theories of consciousness. Cogn Neurosci 2020;12:41–62. 10.1080/17588928.2020.1772214 32663056

[ref8] Fazekas P, Cleeremans A, Overgaard M. A construct-first approach to consciousness science. Neurosci Biobehav Rev 2024;156:105480. doi:10.1016/j.neubiorev.2023.105480 38008237

[ref9] Fink SB . A deeper look at the “neural correlate of consciousness”. Front Psychol 2016;7. 10.3389/fpsyg.2016.01044 PMC4960249 27507950

[ref10] Fink SB . Introspective disputes deflated: the case for phenomenal variation. Philos Stud 2018;175:3165–94. 10.1007/s11098-017-1000-8

[ref11] He BJ . Towards a pluralistic neurobiological understanding of consciousness. Trends Cogn Sci 2023;27:420–32. 10.1016/j.tics.2023.02.001 36842851 PMC10101889

[ref12] Kirkeby-Hinrup A . Interdisciplinary consciousness studies needs philosophers of science. Filosofiska Notiser 2024;11:3–18.

[ref13] Kirkeby-Hinrup A . Quantifying empirical support for theories of consciousness: a tentative methodological framework. Front Psychol 2024;15. 10.3389/fpsyg.2024.1341430 PMC10979646 38558781

[ref14] Kirkeby-Hinrup A, Stenseke J, Overgaard MS. Evaluating the explanatory power of the conscious Turing machine. Conscious Cogn 2024;124:103736. 10.1016/j.concog.2024.103736 39163807

[ref15] Kozuch B . Better bridges: integrating the neuroscience and philosophy of consciousness. Conscious Cogn 2024;126:103774. 10.1016/j.concog.2024.103774 39488884

[ref16] Kvamme TL, Ros T, Overgaard M. Can neurofeedback provide evidence of direct brain-behavior causality? Neuroimage 2022a;258:119400. 10.1016/j.neuroimage.2022.119400 35728786

[ref17] Kvamme TL, Sarmanlu M, Bailey C et al. Neurofeedback modulation of the sound-induced flash illusion using parietal cortex alpha oscillations reveals dependency on prior multisensory congruency. Neuroscience 2022b;482:1–17. 10.1016/j.neuroscience.2021.11.028 34838934

[ref18] Lamme VAF . Separate neural definitions of visual consciousness and visual attention; a case for phenomenal awareness. Neural Netw 2004;17:861–72. 10.1016/j.neunet.2004.02.005 15288903

[ref19] Lamme VAF . Towards a true neural stance on consciousness. Trends Cogn Sci 2006;10:494–501. 10.1016/j.tics.2006.09.001 16997611

[ref20] Lamme VAF . Challenges for theories of consciousness: seeing or knowing, the missing ingredient and how to deal with panpsychism. Phil Transact Royl Soc B 2018;373:20170344. 10.1098/rstb.2017.0344 PMC6074090 30061458

[ref21] Lau, H., & Brown, R. (2019). The emperor’s new phenomenology? The empirical case for conscious experiences without first-order representations. In A. Pautz & D. Stoljar (Eds.), Blockheads! Essays on Ned Block's Philosophy of Mind and Consciousness (pp. 171–97). Cambridge Massachusetts. USA: MIT Press. 10.7551/mitpress/9196.003.0012

[ref22] Lau H, Rosenthal DM. Empirical support for higher-order theories of conscious awareness. Trends Cogn Sci 2011;15:365–73. 10.1016/j.tics.2011.05.009 21737339

[ref23] Mashour GA, Roelfsema P, Changeux J-P et al. Conscious processing and the global neuronal workspace hypothesis. Neuron 2020;105:776–98. 10.1016/j.neuron.2020.01.026 32135090 PMC8770991

[ref24] Melloni L, Mudrik L, Pitts M et al. Making the hard problem of consciousness easier. Science 2021;372:911–2. 10.1126/science.abj3259 34045342

[ref25] Melloni L, Mudrik L, Pitts M et al. An adversarial collaboration protocol for testing contrasting predictions of global neuronal workspace and integrated information theory. PLoS One 2023;18:e0268577. 10.1371/journal.pone.0268577 36763595 PMC9916582

[ref26] Mitchell SD . Integrative pluralism. Biol Philos 2002;17:55–70. 10.1023/A:1012990030867

[ref27] Mitchell, S. D. (2003). Biological Complexity and Integrative Pluralism. Cambridge, UK: Cambridge University Press. 10.1017/CBO9780511802683.

[ref28] Mogensen J, Overgaard M. Reorganization of the connectivity between elementary functions as a common mechanism of phenomenal consciousness and working memory: from functions to strategies. Phil Transact Roy Soc B 2018;373:20170346. 10.1098/rstb.2017.0346 PMC6074082 30061460

[ref29] Negro N . (dis)confirming theories of consciousness and their predictions: towards a Lakatosian consciousness science. Neurosci Conscious 2024;niae012. 10.1093/nc/niae012 PMC10944285 38495333

[ref30] Northoff G, Lamme VAF. Neural signs and mechanisms of consciousness: is there a potential convergence of theories of consciousness in sight? Neurosci Biobehav Rev 2020;118:568–87. 10.1016/j.neubiorev.2020.07.019 32783969

[ref31] Odegaard B, Knight RT, Lau H. Should a few null findings falsify prefrontal theories of conscious perception? J Neurosci 2017;37:9593–602. 10.1523/JNEUROSCI.3217-16.2017 28978696 PMC5628405

[ref32] Overgaard M, Kirkeby-Hinrup A. Finding the neural correlates of consciousness will not solve all our problems. Phil Mind Sci 2021;2. 10.33735/phimisci.2021.37

[ref33] Overgaard M, Sandberg K. The perceptual awareness scale—recent controversies and debates. Neurosci Conscious 2021;2021:niab044. 10.1093/nc/niab044 34925909 PMC8672240

[ref34] Overgaard M, Kirkeby-Hinrup A, Kvamme TL et al. Multisensory Interplay and Theories of Consciousness. Psychol Conscious 2024. Advance online publication. 10.1037/cns0000407

[ref35] Sandberg K, Overgaard M. Using the perceptual awareness scale (PAS). In Overgaard M (ed.), Behavioral Methods in Consciousness Research. USA: Oxford University Press, 2015;181–96. 10.1093/acprof:oso/9780199688890.003.0011

[ref36] Schurger A, Graziano M. Consciousness explained or described? Neurosci Conscious 2022;niac001. 10.1093/nc/niac001 PMC8824704 35145759

[ref37] Signorelli CM, Szczotka J, Prentner R. Explanatory profiles of models of consciousness - towards a systematic classification. Neurosci Conscious 2021;niab021. 10.1093/nc/niab021 PMC8396118 34457353

[ref38] Singhal I, Srinivasan N. Just one moment: unifying theories of consciousness based on a phenomenological “now” and temporal hierarchy. Psychol Conscious 2024. Advance online publication.

[ref39] Storm JF, Klink PC, Aru J et al. An integrative, multiscale view on neural theories of consciousness. Neuron 2024;112:1531–52. 10.1016/j.neuron.2024.02.004 38447578

[ref40] Tsuchiya N, Wilke M, Frässle S et al. No-report paradigms: extracting the true neural correlates of consciousness. Trends Cogn Sci 2015;19:757–70. 10.1016/j.tics.2015.10.002 26585549

[ref41] Yaron I, Melloni L, Pitts M et al. The ConTraSt database for analysing and comparing empirical studies of consciousness theories. Nat Hum Behav 2022;6:593–604. 10.1038/s41562-021-01284-5 35190711

